# Granulome facial de Lever

**DOI:** 10.11604/pamj.2013.16.43.3431

**Published:** 2013-10-07

**Authors:** Naoufal Hjira, Mohammed Boui

**Affiliations:** 1 Hôpital militaire d'instruction Mohammed V, Service de Dermatologie,Rabat, Maroc

**Keywords:** Granulome facial de Lever, masse tumorale, tumeur ulcéro-végétante, Facial granuloma of Lever, tumoral mass, ulcero-vegetating tumour

## Image en médecine

Le granulome facial de Lever se produit le plus souvent chez l'homme de race blanche. Cliniquement, les lésions apparaissent comme des papules érythémateuses, des nodules ou plaques. Le granulome facial est d’étiologie inconnue, mais des facteurs prédisposant sont possibles incluant l'exposition actinique et les traumatismes. La confirmation du diagnostic est faite par une évaluation histologique de la lésion. Sous un épiderme normal, il y a un infiltrat dermique dense, polymorphe, composé de neutrophiles, lymphocytes, les éosinophiles et des plasmocytes. Il n'existe aucun traitement standard pour le granulome facial car aucun traitement n'est efficace. De nombreux traitements ont été essayés y compris les immunosuppresseurs, les lasers à colorant pulsé et l'exérèse chirurgicale. Nous rapportons le cas d'un patient âgé de 73 ans, sans antécédents pathologiques particuliers, présentait depuis un an une lésion papulo-nodulaire para-nasale droite sur laquelle il avait appliqué un traitement traditionnel non précisé. L’évolution était marquée par l'extension de la lésion vers la région pré-auriculaire droite. A l'examen clinique, lésion ulcéro-végétante, érythémato-violine infiltrée, mesurant 15 cm de grand axe, avec œdème important entrainant la fermeture de l’œil droit. Une biopsie cutanée était en faveur d'un granulome facial de Lever. Devant l'importance de l’œdème et de la masse tumorale, une corticothérapie était préconisée, aboutissant à une réduction de la masse tumorale et de l’œdème. Le patient était adressé au centre d'oncologie.

**Figure 1:**
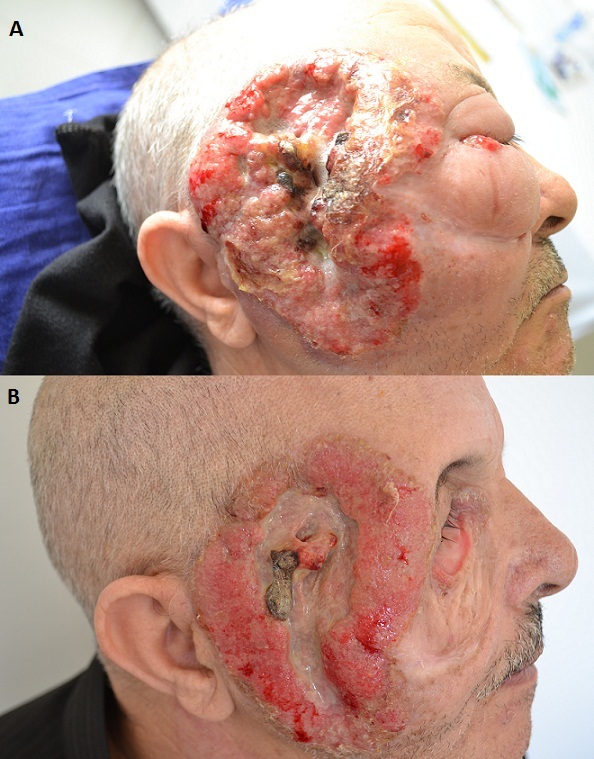
A) Tumeur ulcéro-végétante de la région pré-auriculaire droit; B) Réduction de la masse tumorale après un mois de corticothérapie orale

